# Transarterial embolization for acute lower gastrointestinal bleeding: a retrospective bicentric study

**DOI:** 10.1007/s11547-025-02012-z

**Published:** 2025-04-18

**Authors:** Francesco Tiralongo, Daniele Perini, Luca Crimi, Makoto Taninokuchi Tomassoni, Lorenzo Braccischi, Davide Giuseppe Castiglione, Francesco Modestino, Francesco Vacirca, Daniele Falsaperla, Federica Maria Rosaria Libra, Stefano Palmucci, Pietro Valerio Foti, Francesco Lionetti, Cristina Mosconi, Antonio Basile

**Affiliations:** 1 Radiology Unit 1, Department of Medical Surgical Sciences and Advanced Technologies “GF Ingrassia”, University Hospital Policlinico “G. Rodolico-San Marco”, University of Catania, 95123 Catania, Italy; 2 Department of Radiology, IRCCS Azienda Ospedaliero Universitaria Di Bologna, Via Albertoni 15, 40138 Bologna, Italy; 3 UOSD “IPTRA”, Department of Medical Surgical Sciences and Advanced Technologies “GF Ingrassia”, University Hospital Policlinico “G. Rodolico-San Marco”, University of Catania, 95123 Catania, Italy

**Keywords:** Lower gastrointestinal tract, Gastrointestinal hemorrhage, Embolization, Therapeutic, Angiography, Digital subtraction, Radiology, Interventional

## Abstract

Transcatheter arterial embolization (TAE) represents an effective treatment option for acute lower gastrointestinal bleeding (LGIB). This retrospective, bicentric study evaluated the safety and efficacy of TAE in 77 patients with LGIB. The mean patient age was 68.39 ± 17.54 years, and the mean pre-procedural hemoglobin was 7.87 ± 1.89 g/dL. The most common cause of LGIB was angiodysplasia (36.2%). Pre-procedural computed tomography angiography (CTA) detected active bleeding in 83% of cases. Technical success was achieved in 98.7% of patients, and 30-day clinical success was achieved in 87%. The rebleeding rate was 13%, and the ischemic complication rate was 11.7%. There were no significant associations between clinical success and sex, age, coagulopathy, first-line management, active bleeding signs, culprit vessel, superior mesenteric artery, or time between CTA and digital subtraction angiography. TAE is a safe and effective procedure for LGIB, with high technical and acceptable clinical success rates. It should be considered a standard procedure in select patients, especially when endoscopic treatment is contraindicated or not feasible.

## Introduction

Lower gastrointestinal bleeding (LGIB) is a prevalent clinical condition encompassing a wide array of potential causes, including diverticulosis, angiodysplasia, ischemic colitis, and inflammatory bowel disease [1]. It often manifests as hematochezia, melena, or occult bleeding [1]. LGIB can lead to significant morbidity and mortality, particularly among elderly patients or those with comorbidities [2].

Diagnosis of LGIB often involves clinical assessment, laboratory tests (including complete blood count, coagulation studies, and blood chemistries), and imaging modalities [3]. Colonoscopy is generally considered the first-line diagnostic and therapeutic tool for most patients with LGIB [2, 4, 5]. However, computed tomography angiography (CTA) plays a crucial role in identifying the source of bleeding and guiding further management, especially in severe or ongoing bleeding [2, 4]. CTA can detect active bleeding with high sensitivity and specificity, providing valuable anatomical information about the bleeding site and potential underlying causes [6].

Management of LGIB is tailored to the severity and etiology of the bleeding [7]. For patients with mild or stable bleeding, conservative measures such as fluid resuscitation, blood transfusion (if needed), and correction of coagulopathies may be sufficient [7]. However, in persistent or severe bleeding cases, interventional radiology techniques like transarterial embolization (TAE) have emerged as effective treatment options [7]. TAE involves the selective occlusion of bleeding vessels using embolic agents to achieve hemostasis while minimizing the risk of ischemic complications [8]. Current guidelines recommend TAE for patients with hemodynamically significant LGIB who are not suitable candidates for or have failed endoscopic therapy [2, 4].

This retrospective, bicentric study aimed to evaluate TAE's safety, technical, clinical success, and complication rate in LGIB over a 30-day window.

The secondary outcomes assessed were the detection rate of bleeding on CT and DSA, the use of embolizing materials and their ratio, and the parameters that affected the rebleeding rate.

Lastly, we compared the empirical and target groups about the rebleeding rate and the ischemic rate between the selective and super-selective groups.

## Materials and methods

### Study population and setting

This retrospective, multicenter study evaluated all patients with LGIB bleeding who underwent emergency angiographic evaluation and embolization in two Italian centers between January 2011 and January 2024.

RIS/PACS systems of the participating radiology departments were analyzed to identify all possible cases using the following keywords: embolization, transcatheter embolization, angiography, gastrointestinal bleeding, bleeding, hemorrhage, empirical embolization, and preventive embolization.

The study was conducted according to the tenets of the Declaration of Helsinki. This was a retrospective study, and ethical approval was, therefore, waived.

The medical records of patients identified in the search were retrieved and scrutinized retrospectively.

Patients who could give consent signed a procedural consent form regarding the risks and benefits of the procedure. In cases where patients could not give consent due to the severity of the clinical condition, consent was obtained from relatives where possible.

The final study population included 77 patients with LGIB who underwent angiography study.

Clinical and laboratory data such as pre-interventional hemoglobin, lactate, and INR were obtained from the digital medical records and gathered at a maximum of 12 h before the embolization procedure. Where possible, the etiological causes of bleeding, clinical signs, and first-line management (endoscopic or angiographic) were collected.

All patients suspected of having LGIB underwent contrast-enhanced multi-detector computed tomography (MDCT) before DSA to identify the signs of active bleeding and location.

In patients without signs of active bleeding at CTA, it was decided to proceed with DSA evaluation and eventual embolization because of a patient’s unstable hemoglobin levels, suspected intermittent bleeding, or because of the continuous need for transfusion to maintain acceptable hemoglobin levels (>8.0 g/dL).

### Diagnostic angiography and transarterial embolization

DSA and TAE were performed by interventional radiologists with at least 5 years of experience and radiology residents attending their 3rd or 4th year of training, with anesthesiologic support.

The procedure in the angiography room begins with the placement, under local anesthesia and ultrasound guidance, of a 4-Fr or 5-Fr vascular sheath to maintain stable vascular access. In our institutes, access from the right femoral artery is preferred. A 4–5-Fr typically reverse-curve catheter (Simmons I, Merit Medical, Paris, France) or less commonly 5-Fr guiding catheter Cobra (C1, Medikit, Tokyo, Japan) is then introduced and selective diagnostic angiography, guided by CTA data, is performed at the most likely source of bleeding. Even if the bleeding site could not be identified using SMA or IMA arteriography, arteriography using the SMA or IMA branches (right colic, middle colic, ileocolic, and sigmoid arteries) or selective angiography using the vasa recta or marginal artery was performed using 2.1- to 2.7-Fr microcatheters Direxion (Boston Scientific, Massachusetts, USA) or Progreat (Terumo Corporation, New Jersey, USA). The injection from the microcatheter varies from case to case and can be performed by hand or by the injector.

Signs of active bleeding were evaluated. Findings considered for angiographic proof of bleeding were direct signs of active bleeding (contrast blush extravasation, focal spot of enhancement, hemorrhagic petechiae, and pseudoaneurysm) or indirect signs (vessel cut-off sign or massive vasospasm).

TAE was performed according to standard endovascular embolization techniques, which aim at selective (marginal artery embolization) or super-selective (arteriae rectae embolization) embolization of the bleeding vessel.

A maximum of two vasa recta were embolized to minimize the risk of ischemic complications.

Embolization was carried out via the microcatheter using various embolic agents: pushable or detachable coils; temporary embolic agents such as a re-absorbable gelatin foam; solid particles of PVA (ContourTM PVA Embolization Particles: 150–250 microns, Boston Scientific, Natick, MA, USA) or Embosphere (700–900); adhesive liquid embolic agents as a synthetic biodegradable cyanoacrylate basis glue (Glubran, GEM Italy, Viareggio, Italy); or a combination of these embolic agents.

The choice between the embolic materials was determined according to operator preference and considering the vascular anatomy and the position of the microcatheter within the target vessel.

If no signs of active bleeding at DSA were noted, despite active bleeding in MDCT or endoscopy, empirical embolization of the target vessel was performed in selected cases, guided by the pre-procedural CTA or endoscopic findings, to decrease the arterial bleeding and reduce the risk of recurrent hemorrhage. Other data considered for our study were the target vessel location, the procedural timing, and the time between pre-procedural MDCT and DSA.

### Definitions

Technical success was defined as the complete embolization of all target vessels, without signs of active bleeding on post-embolization angiography.

Clinical success implied the absence of clinical, laboratory, or radiological signs of rebleeding within a 30-day window after the procedure. Clinical failure was considered in patients who presented signs of rebleeding (hemoglobin decrease, hypovolemic shock, or evidence of persistent bleeding at post-procedural CTA examination).

Empirical embolization is defined as the embolization of a target vessel without angiographic evidence of extravasation, typically guided by CTA findings in vessels that appear normal.

Selective embolization was defined as catheterization and embolization of the marginal artery supplying the site of hemorrhage.

Super-selective embolization was defined as catheterization and embolization of the arterial recta supplying the site of hemorrhage.

Procedure-related complications, both minor and major, were classified following the CIRSE classification system, ranging from grade 1 (intra-procedural complication resolved within the same session, with no additional therapy, no post-procedure sequelae, and no deviation from the typical post-therapeutic course) to grade 6 (death) [9].

### Statistics

Statistical analysis was performed using IBM SPSS Statistics version 20 (SPSS Inc., Chicago, IL).

Continuous variables are presented as mean ± SD. Categorical variables are reported as percentages.

Characteristics of the study population were reported as mean (SD), range, and median.

Using independent sample t-tests, the mean values of age, pre-procedural hemoglobin, and time between CT and DSA were compared.

The chi-square test with Yates correction was performed to assess the association between the type of embolization (selective embolization vs. super-selective embolization) and the ischemia rate.

The chi-square test with Yates correction was also performed to compare statistics between target and empirical embolization groups and rebleeding rates.

Univariate binary logistic regression enabled assessing factors associated with recurrent bleeding.

A *p*-value of >0.05 was considered non-statistically significant.

## Results

The initial search retrieved 155 patients. We excluded patients with venous, variceal, or AVM bleeding, patients without DSA, and patients with upper GI bleeding.

The final study population included 77 patients with LGIB who underwent angiography study.

The mean age of the study population was 68.39 ± 17.54 (range 18–96 years). The mean pre-procedural hemoglobin value in our population was 7.87 ± 1.89 g/dL. The pre-procedural INR was 1.29 ± 0.27; pre-procedural lactate was 201.18 ± 90.71 U/L.

We were able to trace the etiology of 47 patients retrospectively. Of these, 49 showed clinical signs of active bleeding (rectorrhagia/melena/severe anemia).

Characteristics of the study population are summarized in Table 1.

**Table 1:** Characteristics of our study population and findings on CTA and DSA

Characteristics	Value
*Age*	
Mean ± SD	68.39 ± 17.54
Range	18–96
Male	44 (57.1%)
Female	33 (42.9%)
*Etiology of bleeding*	
Angiodysplasia	17 (36.2%)
Iatrogenic (post-op)	9 (19.1%)
Diverticulitis	6 (12.8%)
Cancerous	5 (10.6%)
Spontaneous	5 (10.6%)
Meckel diverticula	3 (6.4%)
Traumatic	1 (2.1%)
Pseudomembranous colitis	1 (2.1%)
No information available	30
*Pre-procedural hemoglobin (g/dL)*	
Mean ± SD	7.87 ± 1.89
Range	4.5–9.7
*Localization of bleeding*	
SMA	61 (79.2%)
IMA	16 (20.8%)
*CTA findings*	
Active bleeding	44 (83%)
No proof of active bleeding	9 (17%)
No information available	24
*Angiographic findings*	
Direct/indirect signs of active bleeding	72 (93.5%)
No proof of active bleeding	5 (6.5%)

Pre-procedural CTA demonstrated active bleeding in 44 patients (57%). In 9 patients (12%), CTA did not show signs of active bleeding. Twenty-four patients’ MDCTs were not recorded nor included in our data, since the imaging studies had already been conducted at another hospital facility, or the patient was brought into the angiography room given his medical history.

The most prevalent etiology of LGIB was angiodysplasia, accounting for 36.2% of the bleeding episodes.

The mean delay between angiography and CT was 546.18 min (95% IC 395.14–721.21 min).

The angiographic study showed signs of active bleeding in 72 patients (93.5%); in five patients (6.5%), no angiographic signs of active bleeding were found (Table 1).

TAE was the first-line management choice in 64 out of 77 cases (83.1%), while endoscopy was preferred in 13 (16.9%) cases.

Selective embolization was performed in 55/77 (71.4%) cases, while super-selective embolization was performed in 22 cases (28.6%). Non-selective embolization was not necessary.

TAE was performed in one arterial territory in 76 (92.7%) cases and two arterial territories in 6 (6.3%) cases.

Overall, 82 arteries were embolized, corresponding to a mean of 1.1 per patient.

The embolized arteries were the right colic artery (*n* = 15), ileocolic artery (*n* = 13), superior mesenteric artery in its jejunal section (*n* = 10), superior mesenteric artery in its ileal tract (*n* = 10), sigmoid arteries (*n* = 9), middle colic artery (*n* = 7), cecal artery (*n* = 7), left colic artery (*n* = 5), superior mesenteric artery in its jejunal–ileal section (*n* = 3), and superior hemorrhoidal artery (*n* = 3).

The embolization materials used were absorbable gelatin foam in 34 (44.1%) patients, a combination of Gelfoam and coils in 16 (20.8%) patients, coils in 12 (15.6%) patients, PVA particles in 8 (10.4%), Glubran in 4 (5.2%) patients, a combination of Glubran and gelatin foam in 1 (1.3%) patient, a combination of coils and PVA in 1 (1.3%) patient, and a combination of microsphere and gelatin foam in 1 (1.3%) patient.

The mean procedure time was 54.07 ± 33.40 min (95% CI for the mean 46.35–61.38 min).

The mean duration of the angiographic procedure was less than 60 min in 47 (61%) of the procedures, while it was over 60 min in 30 cases (39%). The duration of the three procedures was not recorded or included in our data.

In five cases, diagnostic angiography did not demonstrate direct signs of active bleeding, so empirical embolization TAE was attempted.

Four of them were treated with super-selective, the latter with selective embolization.

Gelfoam was used in three embolizations; we combined coils and Gelfoam in two cases.

Clinical success was achieved in three cases, while in the other two, due to rebleeding, it was necessary to re-treat with embolization.

Technical success was achieved in 76 out of 77 patients (98.7%). The only complication encountered in the remaining case was the rupture of an AVM.

Clinical success was achieved in 67 patients (75.3%) without further interventions (Table 2).

**Table 2:** The outcome of PTAE

Outcome of percutaneous transarterial embolization	Value *n* (%)
Technical success rate	76 (98.7%)
Clinical success rate	67 (87%)
Complications’ rate	9 (11.7%)
Intestinal ischemia	9

In 10/77 patients (13%), recurrent bleeding occurred within a 30-day window: Nine patients required a second embolization (one 6 h later, one 12 h later, two 24 h later, one 28 h later, one 5 days later, one 6 days later, and two 14 days later), while one patient was treated conservatively, having shown clinical signs of rebleeding 24 h after the TAE, but the following angiography showed no signs of active bleeding.

The proportion of subjects who underwent target or empirical embolization did not differ by rebleeding rate, *X*^2^ (1, *N* = 77) = 1.36, *p* = 0.24.

Sex, age (< or ≥ 70 years old), coagulopathy (INR ≤ or > 1.5), hemoglobin (< or ≥ 8 g/dL), first-line management (endoscopic or angiographic), active bleeding at MDCT and DSA, bleeding site (SMA or IMA), and time between MDCT and DSA were not associated with recurrent bleeding (*p* > 0.05) (Table 3).

**Table 3:** Cross-tabulation or univariate logistic regression did not reveal any associations between the parameters we considered in our study and the incidence of recurrent bleeding

	*B*	S.E	Wald	*df*	Sig	Exp(*B*)
Age (<70; ≥70 y.o.)	77.465	13,859.657	.000	1	.996	4393769956177694000000000000000000.000
Sex	295.257	12,896.240	.001	1	.982	1.692E+128
INR (<1.5; ≥1.5)	328.184	16,981.053	.000	1	.985	3.377E+142
Hb (<8 g/dL; ≥8 g/dL)	− .187	1248.974	.000	1	1.000	.830
First-line management	717.278	39,650.737	.000	1	.986	
Active bleeding at CTA	− 15.133	27,196.457	.000	1	1.000	.000
Active bleeding at DSA	270.059	25,902.700	.000	1	.992	1.929E+117
Culprit vessel	− 53.171	15,413.599	.000	1	.997	.000
Time btw CTA-DSA (min)	− .259	11.369	.001	1	.982	.772
Constant	− 222.482	36,772.182	.000	1	.995	.000

The complication rate (≤30 days) was 11.7%; nine patients experienced post-embolization problems (Table 2). Intestinal ischemia occurred in 9/77 patients, of whom eight underwent emergency surgery.

Hemicolectomies were performed in two cases (one at 24 and one at 48 h), three ileal resections (two at 48 h from TAE and one at 4 weeks from TAE), three jejunal resections (two at 24 h from TAE and one at 1 week from TAE (this patient deceased)), and a sigmoid perforation (which could not be treated because the patient deceased).

A chi-square test of independence showed no significant association between selective or super-selective embolization and ischemic rate, *X*^2^ (1, *N* = 77) = 0.77, *p* = 0.4.

## Discussion

Transarterial embolization (TAE) represents a successful strategy for achieving rapid and safe hemostasis in upper gastrointestinal bleeding (UGIB) [10].

However, a more conservative approach has traditionally been favored for LGIB due to concerns about collateral blood flow limitations and potential bowel ischemia, particularly in the large bowel [11].

Recently, TAE has emerged as a valid tool for diagnosing and treating LGIB, especially in hemodynamic instability, inadequate intestinal preparation, brisk bleeding that hinders endoscopic localization, or failed endoscopic therapy [7, 10].

LGIB is more prevalent in the elderly, with a peak incidence between 63 and 77 years of age [3]. This aligns with our study, where the mean age is 68.39 ± 17.54.

Diverticular bleeding represents the leading cause of LGIB, accounting for 30–50% of cases [12, 13]. Other notable etiologies include angiodysplasia (3–10%), ischemic colitis (2–9%), and infectious or inflammatory bowel disease (6–30%)[14].

Additionally, colonic malignancies, hemorrhoids, and post-surgical bleeding (e.g., post-polypectomy and post-biopsy) can contribute to LGIB [15–17].

In our series, the primary known etiological causes were angiodysplasia (36%), iatrogenic causes (19%), and diverticulitis (13%).

Colonoscopy, with a diagnostic yield of 74–100%, is effective for lower gastrointestinal bleeding and post-operative bleeding [18]. It should be the first-line intervention for hemodynamically stable patients after bowel preparation, unless CTA suggests a small-bowel source [2, 4, 5, 7].

In the diagnostic work-up, CTA has become the method of choice in massive LGIB [19, 20] both as an initial diagnostic test in stable patients with ongoing hemodynamically significant hematochezia [2, 7] and in hemodynamically unstable patients or in a patient who has required more than five units of blood in 24 h [2, 7]. Several studies have investigated the diagnostic performance of CTA in the detection of active bleeding in GIB; Kim et al. [21] showed a diagnostic yield of CTA of 61.3%, with overall sensitivity, specificity, positive predictive value (PPV), and negative predictive value, respectively, of 84.8%, 96.9%, 98.5%, and 72.1%.

The study of Clerc et al. [24] showed lower performances, with an active bleeding detection rate at CTA of 31.3%, but it was significantly higher than endoscopy.

In our study, we report a high detection rate of active bleeding on CTA (83%), believing that pre-procedural CTA represents an effective and fast method for the primary approach to LGIB, both in stable and unstable patients.

The mean time between CTA and DSA was 546 min (range, 40–2880); longer delays weakened the correlation between bleeding on CTA and DSA, reducing technical success [22].

Digital subtraction angiography (DSA) is crucial for diagnosing and managing acute nonvariceal gastrointestinal bleeding [23].

Negative diagnostic rates for DSA range from 24 to 78% [24], but only 6.5% of patients in our study showed no angiographic signs of active bleeding.

Based on the location of the bleeding, the most frequently treated arteries were the right colic artery (18%), ileocolic artery (16%), ileal branches of the superior mesenteric artery (12%), and sigmoid artery (11%), these data confirm that, of all LGIB, bleeding in the small intestine is between 15 and 34% of cases [3, 25, 26].

In this study of 77 patients with LGIB who underwent embolization, selective embolization was performed in 71% and super-selective embolization in 28%. These approaches minimized ischemic complications from non-targeted embolization while balancing the risk of rebleeding [27].

This allowed us to achieve a technical success rate of 98.7%, a 30-day clinical success rate of 87%, and a rebleeding rate of 13%.

These results appear in line with those expressed in a recent meta-analysis, which reported a pooled overall technical success rate and an early rebleeding rate (<30 days) of 97% (95%CI, 95.2–98.5%) and 14.8% (95%CI, 9.9–20.5%), respectively [27].

Nykänen et al. reported that the increasing amount of red blood cells (RBCs) administered before the salvage (*p* = 0.032) and with bleeding originating from the branches of SMA, were significant predictors of rebleeding [26]; as well as Qian-yu et al. [27] considered coagulopathy, inotrope use, and history of cancer as significant predictors of bleeding.

In our series, cross-tabulation or univariate logistic regression did not reveal any associations between clinical success and sex, age, coagulopathy, first-line management, active bleeding signs, culprit vessel SMA, and time between CTA-DSA.

Ischemic complications are common after embolization for LGIB. In the current study, ischemia occurred in 11.7% of patients, with eight requiring surgery and two dying from ischemia.

These values are consistent with the rates reported in the literature, which vary from 7.7 to 26% [25, 26, 28–30], but higher than the pooled overall ischemia rate, the rate of ischemia requiring surgery, and mortality related to ischemia, respectively, of 7.5%, 2.3%, and 0.1% [27].

Liang-Shan et al. [11] did not report patients with intestinal ischemia, as they performed only super-selective embolization—they argued that the embolization was not performed if the catheter tip was not sufficiently peripheral position.

Other studies [26] report how patients with ischemic complications received less selective embolization, underlining the importance of the super-selective approach. Our analysis did not show a significant difference in the ischemia rate between the groups of patients treated with selective and super-selective embolization.

In cases of negative angiography, the use of empiric embolization in LGIBs is little reported in the literature, compared to UGIBs, due to the high risk of ischemic and hemorrhagic complications [31].

Despite this, technological advances and the use of new embolizing materials have led to an initial empirical approach. Only a few studies investigate the effectiveness of this approach, discouraging it in routine practice [26].

Despite this, empiric embolization rebleeding rates appear to be 23.6% on average, comparable to the target approach (21.1%) [27].

In our study, empiric embolization was performed in five patients, with a rebleeding and ischemia rate of 66% and 0%, respectively. There was no significant difference in the rebleeding rate between the groups of patients treated with targeted and empiric embolization.

Recent reports suggest using CBCT-guided embolization using navigation software tools in cases with negative angiographic findings [20, 26].

In our experience, empiric embolization in LGIB is discouraged due to the high risk of rebleeding, except in selected cases where pre-procedural CTA or endoscopic examination can guide embolization.

There is no evidence to support using a specific embolic agent to treat LGIB [31]. Embolization can be performed with gelatin sponges, microcoils, particles, and, more recently, glue, although the operator makes the final decision based on his experience and compliance [20].

Microcoils have the advantages of being radiopaque, low cost, easy to use, and able to provide super-selective embolization. The surgeon can palpate them, guiding him in any intestinal resection. However, their efficacy depends on the patient's coagulation status [20, 26, 27].

Cyanoacrylates are liquid adhesive agents whose monomers polymerize in contact with blood; they are very effective in controlling bleeding.

However, they have a high risk of non-target embolization and catheter entrapment, as they are not controllable and require extensive operator experience [20, 27].

When comparing microcoils and cyanoacrylates, Qian-yu et al. [27] reported a lower rate of rebleeding in the latter (pooled rebleeding rate of 9.3% vs. 20.8%), compared to a lower rate of ischemia in patients treated with microcoils (9.7% vs. 4.0%).

Although Gelfoam is generally considered safe for treating LGIB, its use has limitations. It may fail to achieve complete arterial occlusion, leading to a higher risk of recurrent bleeding than other embolic agents. Additionally, small gelatin sponge particles can migrate distally into collateral vessels, increasing the risk of intestinal ischemia [11].

PVA particles represent an effective embolizing agent because they achieve rapid and complete embolization, regardless of the diameter of the artery; however, they are associated with an increased risk of intestinal infarction, as they are difficult to handle and cannot be targeted precisely and can reflux in non-target arteries, traveling distally beyond the level of collateralization [11, 20].

In our experience, the most used embolizing materials were gelatin sponges (44%), a combination of gelatin sponge and coils (21%), and coils (16%).

Non-adhesive liquid embolizing agents, such as the ethylene–vinyl alcohol copolymer, Onyx® (Medtronic), have also begun to be more widely used in peripheral embolizations, due to their properties of filling the vascular lumen, without adhering to the vessel in contact with the blood, thus providing a controlled release, progressive solidification, and high vascular penetration; however, they have long preparation times and are very expensive [20, 27, 32, 33].

Initial experiences with these embolic agents have shown that super-selective embolization with Onyx is feasible and safe, with a rebleeding rate of 10% and without cases of ischemia [34].

Hiraki et al. [35] recently reported using an imipenem/cilastatin (IPM/CS) mixture as an embolic agent in TAE for acute LGIB. They injected the embolic agent from a marginal artery near the bleeding site and reported technical and clinical success rates of 100% and 83.3%, respectively. This suggests that if super-selective embolization is impossible, IPM/CS is a viable option for stopping the bleeding in cases of acute LGIB.

We are aware that this study had some limitations, particularly the retrospective design. We could not evaluate whether patients showed signs of shock, use of inotropes, or red blood cell transfusion before performing the procedure, as these data were not available in our digital medical records.

Another study limitation was the lack of long follow-up to evaluate long-term survival.

Our study's strength lies in the large sample size and multicenter nature.

## Conclusion

TAE is a safe and effective therapeutic option for treating LGIB, with a high technical success rate and acceptable clinical success rate.

TAE should be considered a standard procedure in selected patients, especially if endoscopic treatment is contraindicated or cannot be performed.

Yet, thanks to the most recent technological innovations and their good results, it can also be considered a first-line therapeutic choice.

We discourage empiric embolization in LGIB due to the high risk of rebleeding, except in cases where embolization can be guided by a pre-procedural CTA or endoscopic examination.
