# Correction: Transarterial embolization for acute lower gastrointestinal bleeding: a retrospective bicentric study

**DOI:** 10.1007/s11547-025-02024-9

**Published:** 2025-05-20

**Authors:** Francesco Tiralongo, Daniele Perini, Luca Crimi, Makoto Taninokuchi Tomassoni, Lorenzo Braccischi, Davide Giuseppe Castiglione, Francesco Modestino, Francesco Vacirca, Daniele Falsaperla, Federica Maria Rosaria Libra, Stefano Palmucci, Pietro Valerio Foti, Francesco Lionetti, Cristina Mosconi, Antonio Basile

**Affiliations:** 1 Radiology Unit 1, Department of Medical Surgical Sciences and Advanced Technologies “GF Ingrassia”, University Hospital Policlinico “G. Rodolico-San Marco”, University of Catania, 95123 Catania, Italy; 2 Department of Radiology, IRCCS Azienda Ospedaliero Universitaria Di Bologna, Via Albertoni 15, 40138 Bologna, Italy; 3 UOSD “IPTRA”, Department of Medical Surgical Sciences and Advanced Technologies “GF Ingrassia”, University Hospital Policlinico “G. Rodolico-San Marco”, University of Catania, 95123 Catania, Italy

**Correction to: La radiologia medica** 10.1007/s11547-025-02012-z

In the original publication, second author name is published wrongly as Daniele Perrini it should be corrected as Daniele Perini.

Original article has been updated.

